# Synthesis, Spectroscopic Properties and Redox Behavior Kinetics of Rare-Earth Bistetrakis-4-[3-(3,4-dicyanophenoxy)phenoxy]phthalocyaninato Metal Complexes with Er, Lu and Yb

**DOI:** 10.3390/molecules26082181

**Published:** 2021-04-10

**Authors:** Dmitry A. Erzunov, Anna A. Botnar, Natalia P. Domareva, Tatiana V. Tikhomirova, Arthur S. Vashurin

**Affiliations:** Department of Inorganic Chemistry, Ivanovo State University of Chemistry and Technology, 153000 Ivanovo, Russia; anna.filippova96@gmail.com (A.A.B.); domarevanat@gmail.com (N.P.D.); tararjkina@mail.ru (T.V.T.); asvashurin@mail.ru (A.S.V.)

**Keywords:** phthalocyanines, rare-earth complexes, synthesis, spectroscopy, redox activity

## Abstract

Novel bistetrakis-4-[3-(3,4-dicyanophenoxy)phenoxy]phthalocyaninato of complexes erbium, lutetium and ytterbium were synthesized using a template fusion method to prevent any polymerization process. The complexes were separated from the reaction mixtures and characterized by NMR, IR and electron absorption spectroscopy. The spectroscopic properties of the metal phthalocyaninates in chloroform, acetone and tetrahydrofuran were studied. The regular bathochromic shift in the Er–Yb–Lu series was determined. In acetone medium all the complexes obtained were found to exist in an equilibrium state between neutral and reduced forms. The linearity of Lambert-Bouger-Beer curves makes it possible to study the kinetics of redox processes in the presence of phenylhydrazine and bromine. The lutetium complex showed better reducing properties and turned fully into the reduced form, while the erbium and ytterbium ones changed only partially. Upon oxidizing all the phthalocyaninates transformed into a mixture of oxidized and neutral-radical forms. The extinction coefficients and effective redox constants were calculated.

## 1. Introduction

Phthalocyanines are widely used in many areas of activity, such as catalysis [1,2], light industry [3,4], medicine [5,6], linear and nonlinear optics and energy storage devices [7,8,9]. Such a widespread usage is associated with the high stability of these molecules, the presence of intense light absorption in the visible range, the flexibility of the design of structures [10,11,12], the possibility of introducing practically any metal atom into the central cavity [13,14,15] and the possession of significant coordination properties. The scope of usage directly depends on the structure of the molecule, namely on the nature of peripheral and non-peripheral substituents, the presence or absence of a central metal atom. The environment in which the connections are used is also strongly influenced. For example, unsubstituted phthalocyanine is practically insoluble in most organic media, which greatly limits the scope of its application.

Double-decker phthalocyaninates of rare-earth elements are of particular interest to scientists due to the special structure of molecules, namely, the presence of an unshared electron delocalized between two aromatic fragments of ligands, which is responsible for the manifestation of atypical physical and chemical properties [16,17,18], such as electrochromism [19,20], nonlinear optical absorption of light [9,21,22], redox transitions [23]. The properties of such structures strongly depend on the central metal atom, which is responsible for the distance between two coordinated phthalocyanine ligands, as well as the nature of substituents which also make a contribute.

In the literature, there are a sufficient number of examples of the synthesis and study of the properties of peripherally and non-peripherally substituted double-decker phthalocyaninates of rare-earth elements with alkyl-, aryl-, etc. functional groups [24,25]. However, often these types of substitution lead to strong aggregation of molecules, which adversely affects all the properties they exhibit. The introduction of aryloxy- fragments into the structure of the phthalocyanine ring followed by the formation of a sandwich structure has been described to a limited extent; there are only a few works devoted to this topic [26]. Despite this, as we previously shown [27,28] this type of substitution for mono-complexes provides excellent solubility for compounds along with intense light absorption and aggregation stability, due to the presence of aryl- fragments that provide solubilization and oxygen bridges that impart spatial flexibility of molecules, which together can facilitate the further use of materials based on these compounds. The introduction of cyano- groups to the periphery leads to difficulties in the synthesis and purification of complexes; therefore, there are practically no works on this topic in the literature. However, the presence of terminal cyano- groups on the periphery greatly simplifies the preparation of functional materials based on the described ones due to the emerging possibility of simple and stable binding of molecules to any carrier.

This work describes the preparation of new double-decker bistetrakis-4-[3-(3,4-dicyanophenoxy)phenoxy]phthalocyaninates with erbium, ytterbium and lutetium. The complexes were obtained as a result of template fusion, leading to the suppression of the course of polymerization processes, characterized and studied for their spectroscopic and redox properties.

## 2. Results and Discussion

### 2.1. Synthesis of 4,4’-[1,3-Phenylenebis(oxy)]phthalodinitrile

**Scheme 1:**
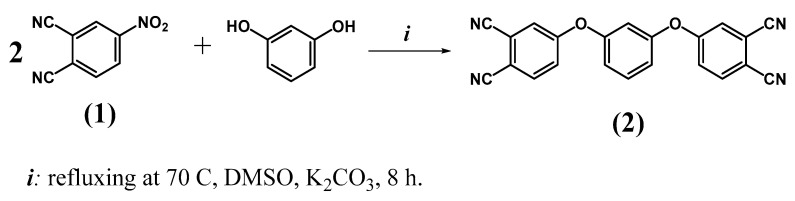
Synthesis of 4,4’-[1,3-phenylenebis(oxy)]phthalodinitrile (**2**).

At the first stage, 4,4’-[1,3-phenylenebis(oxy)]phthalodinitrile (**2**) was obtained by nucleophilic substitution in 4-nitrophthalonitrile (**1**) in a reaction with resorcinol (Scheme 1) following a modified version of the experimental protocol reported in [27]. The reaction proceeded in anhydrous DMSO for 8 h, and its progress was monitored by thin layer chromatography on SiO_2_ using ethanol as an eluent. After the reaction, the compound was precipitated with a 0.1 mol/L hydrochloric acid solution, filtered on a Schott filter, and washed with distilled water, an aqueous acid solution (HCl), and water to neutral pH. Excess solvent was removed under vacuum. The compounds were purified by recrystallization from ethanol. The compound yield was 82%.

Compound **2** was characterized by IR, NMR spectroscopy and MALDI-TOF spectrometry. In the IR spectrum, all characteristic vibrations inherent in the described structure are observed, such as 3088–2850 cm^−1^ (C*_ar_*-H); 2243 cm^−1^ (C≡N); 1586–1389 cm^−1^ (C*_ar_*=C*_ar_*); 1256 cm^−1^ (Ar–O–Ar). The degree of completion of the nucleophilic substitution reaction was also monitored using IR spectroscopy. The spectrum of compound **2** after purification does not contain symmetric (1340–1350 cm^−1^) and asymmetric (1560–1565 cm^−1^) vibrations of the nitro groups of the initial 4-nitrophthalonitrile (**1**). Also, the appearance of a band at 1256 cm^−1^ is observed, which is responsible for the vibrations of the Ar-O-Ar group resulting from the reaction. The high resolution mass spectra represent a signal at *m/z* 362.11, corresponding to the structure of phthalodinitrile **2**. The structure was confirmed by the position, shifts, and signal multiplicity in the ^1^H- and ^13^C-NMR spectra.

### 2.2. Synthesis of f-Metal Phthalocyaninates

**Figure 1:**
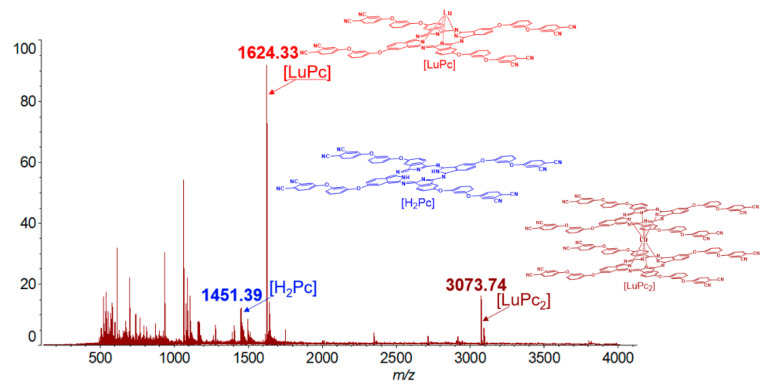
HR MALDI-TOF mass spectrum of reaction mixture after template fusion reaction with lutetium(III) acetate (CHCA as matrix).

**Scheme 2:**
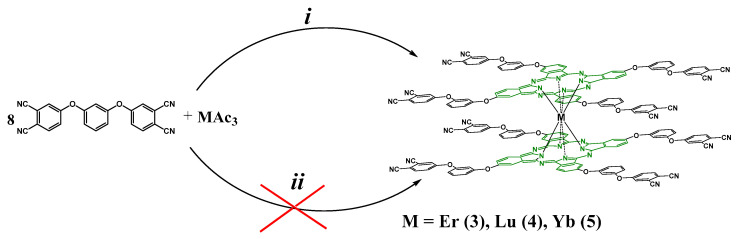
Synthetic routes of erbium (**3**), lutetium (**4**) and ytterbium (**5**) bistetrakis-4-[3-(3,4-dicyanophenoxy)phenoxy]phthalocyaninato complexes obtaining. *i*: template fusion in ceramic crucible in absence of solvent, 190 °C, 20–30 min; *ii*: tetramerization in high-boiling solvent (*iso*-amyl alcohol), reflux, 24 h.

There are several methods for obtaining metal complexes of phthalocyanines. One of the most commonly used is condensation in a high boiling solvent. For these purposes, 4,4’-[1,3-phenylenebis(oxy)]phthalodinitrile (**2**) was mixed with anhydrous erbium(III) acetate in a molar ratio of 8:1 in dry *iso*-amyl alcohol medium in the presence of DBU at refluxing (Scheme 2). The reaction progress control was carried out using thin layer chromatography (SiO_2_, chloroform as eluent). Unfortunately, even after 15 min after the start of the reaction, the target compound was not detected, at the same time, a high degree of polymerization of the reaction mass was observed and the final product was black crystals, insoluble in commonly used organic solvents. Varying the reaction temperature, solvent and component ratio did not give any result. Thus, a different approach was chosen, consisting in cyclic tetramerization in the absence of a solvent (Scheme 2). These conditions are more stringent, which has a detrimental effect on the yields of complexes; however, they open up the possibility of direct preparation of compounds, as we have shown earlier [29]. In a ceramic crucible 4,4’-[1,3-phenylenebis(oxy)]phthalodinitrile (**2**) and anhydrous lanthanide (III) acetate in a molar ratio of 8:1 were placed, then urea was added to facilitate the transfer of the mixture into the alloy. The reaction proceeded at 190 °C for about 20–30 min (depending on the metal), after which the alloy was cooled to room temperature and dissolved in chloroform, followed by filtration in order to separate it from the impurities of the polymer composition and salt residues. The mass-spectrum of the filtrate showed the presence of three main compounds in the mixture, namely, metal-free tetrakis-4-[3-(3,4-dicyanophenoxy)phenoxy]phthalocyanine, lantanide tetrakis-4-[3-(3,4-dicyanophenoxy)phenoxy]phthalocyaninato monocomplex and target sandwich lantanide bistetrakis-4-[3-(3,4-dicyanophenoxy)phenoxy]phthalocyaninato complex (Figure 1).

To isolate the desired compound the solvent was distilled off under vacuum and the mixture was preliminarily purified by column chromatography on silica gel M60, eluting with a gradient of chloroform with ethanol (from 0 to 15 vol.%). Final purification was performed by gel permeation chromatography on Bio-Beads S-X1 gel with a mixture of ethanol (2.5%) in chloroform.

**Figure 2:**
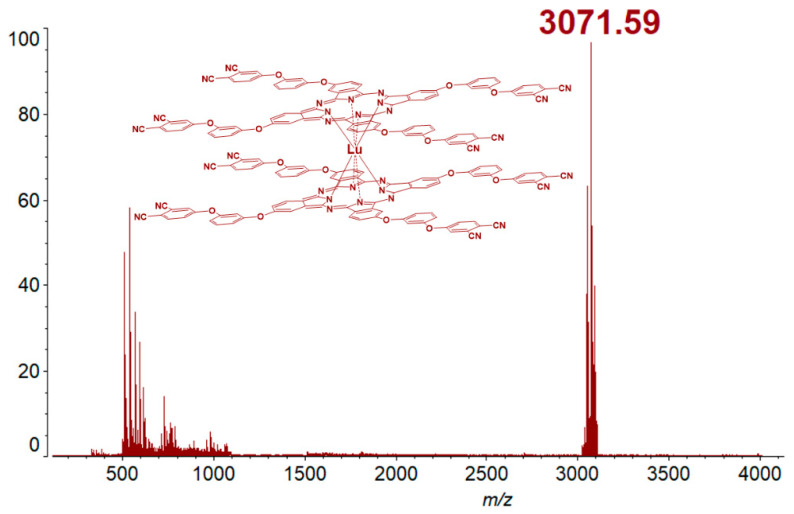
HR MALDI-TOF mass-spectrum of bistetrakis-4-[3-(3,4-dicyanophenoxy)phenoxy]phthalocyaninato lutetium (**4**) (CHCA as matrix).

Compounds **3–5** were identified using IR, NMR spectroscopy, MALDI-TOF mass spectrometry data. The mass-spectrum of the compound after the two-step purification no longer contained the signals of the monocomplex and metal-free phthalocyanine, as previously shown (Figures S1 and S2). There is only an intense band corresponding to the target complex (Figure 2). This partly proves successful cleaning of the compound.

**Figure 3:**
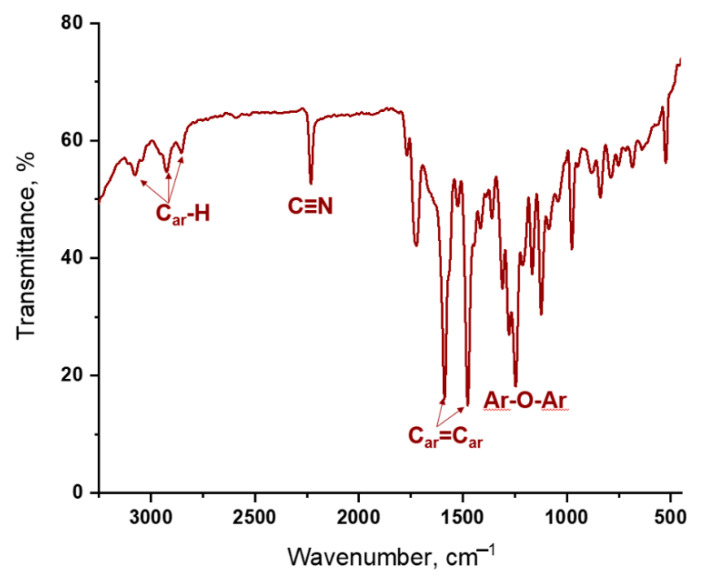
FT-IR spectrum of bistetrakis-4-[3-(3,4-dicyanophenoxy)-phenoxy]phthalocyaninato ytterbium (**5**).

The IR spectra of complexes **3–5** contain the entire set of characteristic bands (Figure 3). Besides, a phthalocyanine metal complex obtained from corresponding dinitrile has more intensive band responding to vibrations of C≡N group (~2232 cm^−1^).

**Figure 4:**
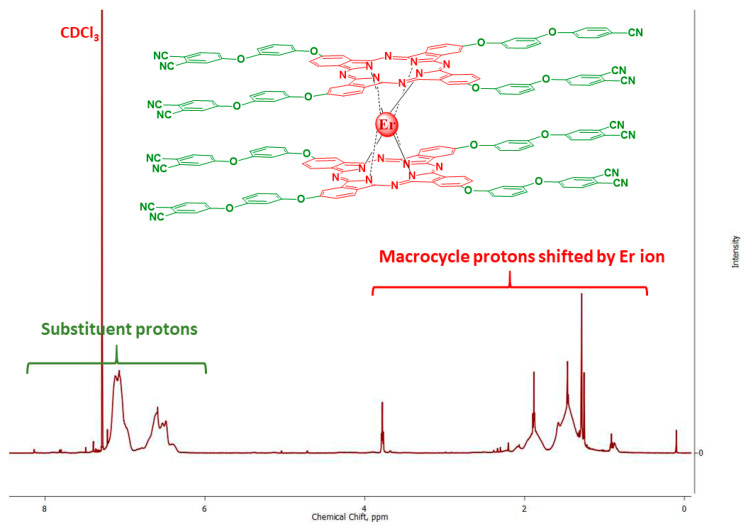
^1^H-NMR spectrum of bistetrakis-4-[3-(3,4-dicyanophenoxy)phenoxy]phthalocyaninato erbium (**3**) in CDCl_3_ (the atoms most affected by the central metal atom are highlighted in red, the least in green).

^1^H-NMR spectra were also recorded for all complexes (Figure 4, Figures S3 and S4). For paramagnetic erbium and ytterbium, a broadening of all manifested signals and shifts of protons of macrocyclic fragments to the region of a strong field were observed under the influence of proximity to a metal atom. An interesting fact is that the signals of the protons of the peripheral substituents were not shifted and appeared in the aromatic region, which is probably due to their relative remoteness from the complexing atom; however, the effect of the paramagnetic metal broadens the described signals as well (Figure 4).

### 2.3. Spectroscopic Properties of Rare-Earth Metal Phthalocyaninates

Due to their unique structure, namely the presence of an extended conjugated aromatic ring, compounds of the phthalocyanine class exhibit strong light absorption in the visible region. This fact makes it possible to study the properties of the described compounds using electronic absorption spectroscopy. The electronic absorption spectrum of phthalocyanine ligands is represented by a band in the near ultraviolet (*B*-band, ~350 nm), which is responsible for deep electronic transitions and an intense split band at 600–700 nm (*Q*-band) with two vibrational satellites, which is responsible for a purely electronic π-π* transition. This structure is described by the *D*_4*h*_ symmetry group. Ongoing to metal complexes, an increase in symmetry to *D*_2*h*_ (in the case of monocomplexes) is observed, accompanied by the convergence of the energies of the HOMO and LUMO, which manifests itself in the degeneracy of the *Q-*band, i.e., merging the two components together. Sandwich bis-phthalocyaninates in the electronic spectrum have additional characteristic bands at 450 nm (broadened shoulder) and 900 nm, which appear as a result of electron delocalization between two aromatic systems in the neutral-radical form of the metal complex.

However, phthalocyanine itself and its complexes have very low solubility in most solvents, which complicates both the study of properties and the usage of materials based on them. To impart solubility to the periphery, one or another organic substituent is usually introduced, the nature of which affects the properties of the molecule. In this work, complexes of a sandwich structure with 3-(3,4-dicyanophenoxy)phenoxy- substitution are presented. The presence of several aromatic fragments improves the spectroscopic properties of the compounds (influencing the solubility and the ability to absorb light) and the connection of aryl- fragments through oxygen bridges makes the peripheral substituents flexible and mobile, which prevents the aggregation of molecules in the liquid phase (since aggregation adversely affects all manifested compounds properties).

**Figure 5:**
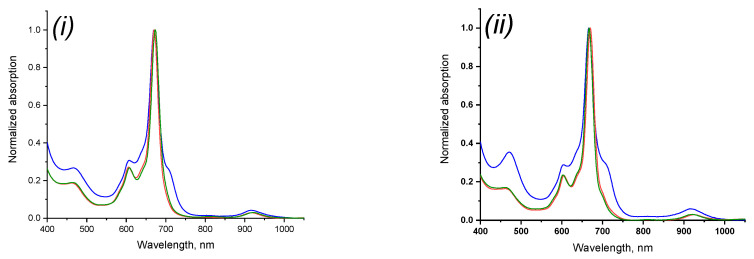

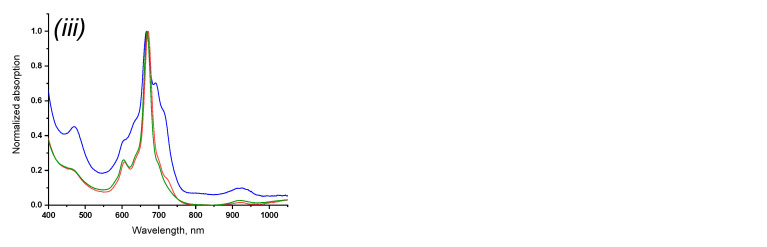
Normalized electronic absorption spectra of **3** (***i***), **4** (***ii***) and **5** (***iii***) in acetone (blue curve), THF (red curve), chloroform (green curve).

The introduction of substituents made it possible to study the spectroscopic properties of the obtained phthalocyaninates of rare-earth metals. The electronic absorption spectra of compounds **3–5** in acetone, tetrahydrofuran and chloroform were obtained (Figure 5). Chloroform corresponded to the most bathochromic shift, while for acetone and tetrahydrofuran the absorption maxima of light in the *Q*-band were approximately equal, which, on the one hand, may be associated with an increase in polarity in the series, and, on the other hand, with a lower coordination the ability of chloroform in the studied series of solvents, which causes less solvation of the coordination center of the molecule and, as a consequence, its availability (Table 1).

A regular bathochromic shift of the absorption spectra in the Er–Yb–Lu series was also observed for all media studied. This fact can be associated with an increase in the atomic mass of the central metal atom accompanied by a change in orbital energies.

The absorption spectra of compounds **3–5** in chloroform and tetrahydrofuran had the typical view for the neutral-radical form, characterized by the presence of a shoulder at 450 nm and an additional low-intensity absorption band at 900 nm. However, in acetone, the spectra for all complexes turned out to be broadened and the *Q*-band was characterized by an atypical form for sandwich phthalocyaninates due to the presence of additional absorption (some type of a shoulder) in the long-wavelength part. Using thin-layer and sorption column chromatography it was determined that in the acetone medium compounds **3–5** exist in the equilibrium state of the neutral-radical and reduced forms, which were further separated and analyzed. The origin of this equilibrium remained unknown. In other studied solvents, the equilibrium described was not observed.

**Figure 6:**
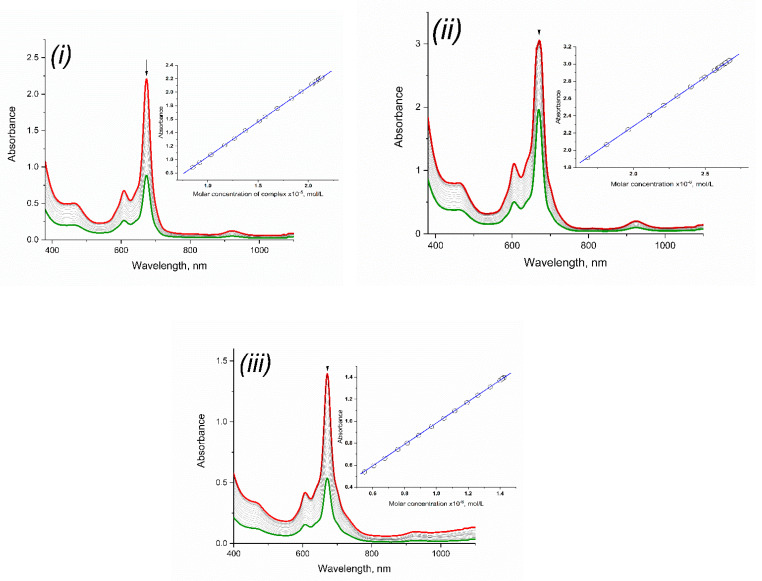
Dilution of solutions of **3** (***i***), **4** (***ii***) and **5** (***iii***) in CHCl_3_ at concentration range 2.04 × 10^−5^ ÷ 8.51 × 10^−6^ mol/L. Insertion: Lambert-Bouguer-Beer curves.

For the complexes obtained the aggregation properties in chloroform and tetrahydrofuran were studied. In view of the occurrence of the equilibrium processes described earlier in the acetone solution the studies were not carried out. It was determined that in the studied concentration range compounds **3–5** exist in monomeric form in chloroform and tetrahydrofuran, which made it possible to carry out further studies of the redox properties of the complexes and to determine the kinetic characteristics of the processes (Figure 6).

### 2.4. Redox Equilibriums in the Solutions of Rare-Earth Metal Phthalocyaninates

Double-decker macrostructures are usually formed in the form of stable neutral radicals [Pc^−^Ln^3+^Pc^−^]^0^—the “green” form, where, when examining the diagram of molecular orbitals, there is an unpaired electron on a single occupied molecular orbital. One of the most important properties of double-decker complexes of phthalocyanine with lanthanides is the ability to enter kinetically controlled redox processes with the participation of oxidizing/reducing agents. Due to the possibility of accepting or transferring an electron, various redox forms can exist in solution: anionic “blue” [(Pc^2−^)_2_Ln^3+^]^−^ and cationic “red” [(Pc^–^)_2_Ln^3+^]^+^ ones [29]. It should be noted that there are few data in the literature on kinetically controlled redox reactions involving diphthalocyanines [30,31]. Further, the kinetic behavior for cyano-substituted sandwich phthalocyanines is considered depending on the nature of the lanthanide ion. An aprotic solvent tetrahydrofuran was used as a solvent, in which a high solubility of the studied macrocycles is observed.

**Figure 7:**
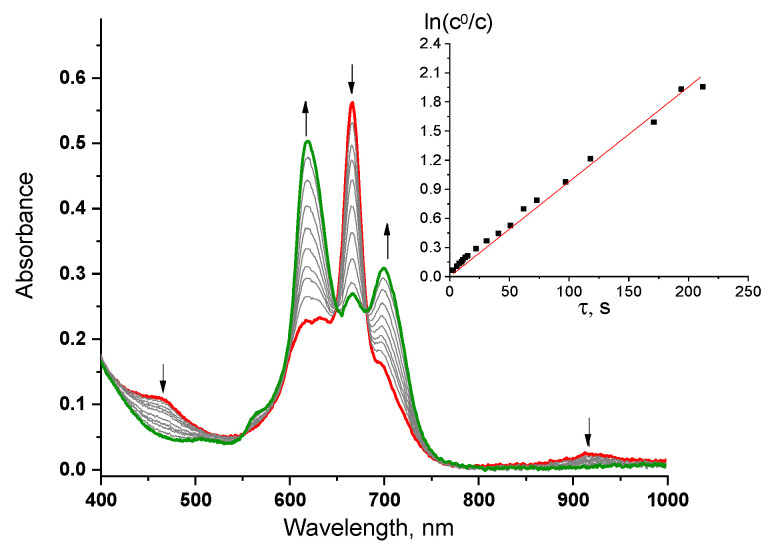
Changes in UV-vis for **4** in a THF solution (c = 1.2 × 10^−5^ mol/L) in the presence of phenylhydrazine (c = 3.3 × 10^−4^ mol/L) for 4 min (red curve–the initial spectrum, green–the final one). Insertion: *ln(c^0^/c)* versus the reaction time of LuPc2 with phenylhydrazine.

Figure 7 shows the changes in the UV-vis for lutetium complex in the presence of a reducing agent represented by phenylhydrazine. In the electronic spectra a simultaneous decrease in the intensity of absorption bands at 465 nm, 667 nm, and 915 nm, characteristic of the existence of a macrocycle in a neutral-radical form and an increase in intensity at 620 nm and 700 nm, which is characteristic of the reduced anionic form, are recorded with time. The formation of the reduced form for **4** obeys the pseudo first kinetic order with respect to phthalocyanine (insert in Scheme 1), as evidenced by the rectilinear character of the dependence of *ln(c^0^/c)* on *τ*.

**Figure 8:**
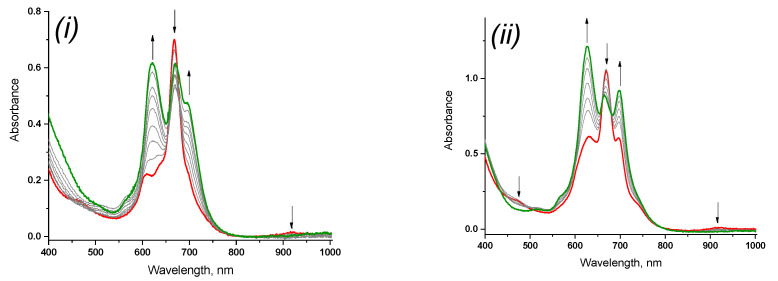
Changes in the UV-vis for **5** (***i***) at (c = 2.1 × 10^−5^ mol/L) and **3** (***ii***) (c = 1.2 × 10^−5^ mol/L) in a THF solution in the presence of phenylhydrazine 3 × 10^−4^ mol/L) min (red curve—the initial spectrum, green—the final one).

Considering similar reactions for **3**, **5** it should be noted that base on final view of UV-vis spectra the process of transition to the anionic form of sandwich phthalocyanines does not proceed completely: the contribution of both two forms to the *Q*-band is observed (Figure 8*i***,** *ii*) (other conditions being equal). This fact can be associated with the ionic radius of the lanthanides. The lutetium ion has the smallest radius, which leads to a stronger π-π-interaction of two phthalocyanine ligands and, as a consequence, a more complete course of the redox process with the addition of a reducing agent, while the effective rate constant of the process was 3.9 × 10^−3^ s^−1^. On the other hand, for ytterbium and erbium ions, due to an increase in the ionic radius and an increase in the distance between macrocycles, the reduction of diphthalocyanine proceeds with more difficultly with the formation of a mixture of neutral and anionic forms with close rate effective constants 3.5 × 10^−3^ s^−1^ and 2.3 × 10^−3^ s^−1^ (Table 2).

**Figure 9:**
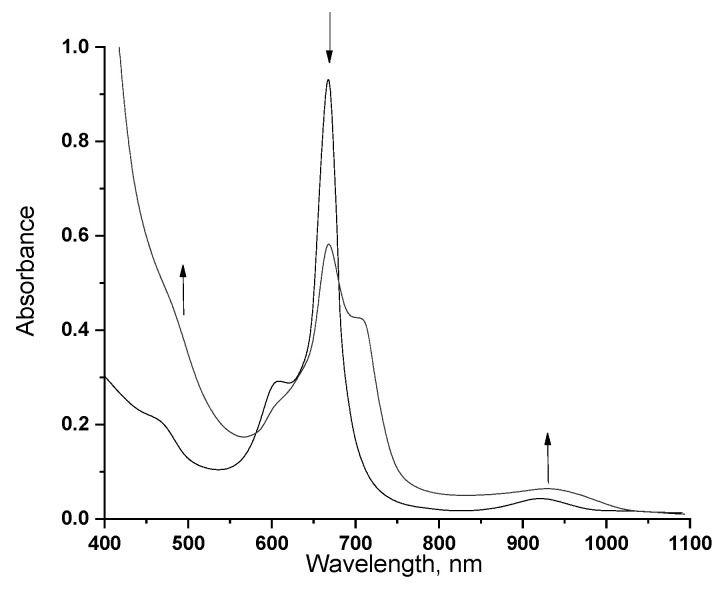
Changes in UV-vis for **4** in a THF solution (c = 1.6 × 10^−5^ mol/L) in the presence of bromine (c = 3.9 × 10^−3^ mol/L).

The experimental results show that the oxidation of the studied sandwich phthalocyaninates by bromine (c = 3.9 × 10^−3^ mol/L) in a THF solution occurs extremely quickly for all the systems under study, which did not allow calculating the kinetic parameters of the process. By addition of bromine to the solutions of sandwich macrocycles in UV-vis, an instantaneous change in the spectra is observed: an increase in intensity in the region of 450 nm and 900 nm, which is typical for oxidized forms, while the optical density of the *Q*-band decreases and an absorption maximum appears at 710 nm (Figure 9). Exposure of these solutions for 2 h leads to the destruction of phthalocyanine complexes with the formation of colorless low molecular weight products. It should be noted that the maximum in the region of 900 nm is much less intense than in the presented works [29,32,33]. This fact can be explained by the incompleteness of the oxidizing process, which leads to the partial presence of neutral forms of double-decker phthalocyanines. Similar changes were observed for **3**, **5**.

Thus, when phenylhydrazine is added to solutions of the lutetium complex, a complete transition from the neutral-radical to the reduced form is observed, while for **3** and **5** it is partial. For all studied macrocycles, oxidation with bromine leads to the formation of a mixture of neutral-radical and oxidized forms. The ability of sandwich molecules to change spectroscopic characteristics under the action of agents of various natures is promising for creating prototypes of sensor devices.

## 3. Materials and Methods

### 3.1. Equipment and Reagent

Elemental analysis of the synthesized compounds was carried out on a CHNS-OFlashEA, 1112 series analyzer (Thermo Quest, Milan, Italy). IR spectra were recorded on IRAffinity-1S spectrometer (Shimadzu, Kyoto, Japan) in the region of 450–4500 cm^−1^. Electronic absorption (UV-vis) spectra were obtained using UNICO2800 (United Products and Instruments, New Jersey, USA) and AvaSpec-ULS2048CL-EVO (Avantes, Louisville, USA) spectrophotometers at the wavelength range of 190–1100 nm in DMF, acetone and CHCl_3_ utilizing quartz cuvettes of optical path length equal to 1 cm. NMR spectroscopy were studied on an AVANCE 500 spectrometer (Bruker, Hamburg, Germany) with TMS as inner standard, registering ^1^H nuclei at 500 MHz in CDCl_3_. Mass-spectra were obtained by means of an Axima Confidence time-of-flight mass-spectrometer (MALDI-TOF MS) (Shimadzu, London, UK). Samples of approximately 10^−3^ mol/L concentration were stored in tetrahydrofuran and CHCA (alpha-cyano-4-hydrohycinnamic acid) was used as a matrix.

Purification of the complexes obtained was performed utilizing column (on silica gel 60, 200–400 mesh) and gel permeation (on Bio-Beads S-X1) chromatography. The homogeneity of the products was tested at each step by TLC (on SiO_2_).

4-Nitrophthalonitrile (99%, Sigma-Aldrich, Missouri, USA), chloroform (99%), ethyl alcohol (99%), acetone (99%), tetrahydrofuran (THF) (99%), dimethylsulfoxide (DMSO) (99%), hydrochloric acid (33%), resorcinol (98%) were applied without additional purification. Potassium carbonate and acetate of lanthanides were dehydrated right before the experiments.

### 3.2. The Study of Kinetic Properties

Phenylhydrazine (Sigma Aldrich) and bromine (Merck, Darmstadt, Germany) were used for the reduction and oxidation of solutions of sandwich-complexes, respectively.

To carry out kinetic studies, a freshly prepared solution of diphthalocyanine was placed in a thermostated cell at 298 K of the spectrophotometer. The study of the kinetics in a THF solution was carried out in quartz cuvettes with an absorbing layer thickness of 1 cm. Preliminarily thermostated solutions of diphthalocyanine and an oxidizing/reducing agent were mixed in the cuvette, then the optical density of the solutions was measured at intervals. The rate of the redox process was determined by the decrease in the optical density of macrocycle solutions at λ_max_ for the neutral-radical form. The differences in the absorption maxima of the neutral-radical, reduced and oxidized forms made it possible to determine the current and final concentration of the studied diphthalocyanines:

(1) $$c=c^{0}\left(A_{\tau }-A_{\infty }\right)/\left(A_{0}-A_{\infty }\right)$$

where *c* is the current concentration, *c*^0^—the initial concentration, *A_τ_*—the optical density of the solution at the moment of time *τ*, *A*_∞_—the optical density of the solution after the completion of the reaction, *A*_0_—the optical density of the solution at the initial moment of time.

The measurements were carried out under the conditions of a pseudo-first order reaction; therefore, the effective rate constant was calculated using the equation:

(2) $$k_{obs}=\left(\frac{1}{\tau }\right)\ln\left(A_{\tau }-A_{\infty }\right)/\left(A_{0}-A_{\infty }\right)$$

where *τ*—the time from the start of the reaction.

### 3.3. The Study of Aggregation Properties

The aggregation properties have been studied in a number of organic solvents such as acetone, chloroform, tetrahydrofuran. The concentrated phthalocyanine solution was diluted with an appropriate solvent and the dependence of the optical density of the absorption maximum on the concentration of the complex in the solution was plotted (checking the linearity of the Lambert-Bouguer-Beer curve).

### 3.4. Synthesis

#### 3.4.1. Synthesis of 4,4’-[1,3-Phenylenebis(oxy)]diphthalonitrile

Resorcinol (0.09 g, 0.86 mmol), potassium carbonate (0.12 g, 0.86 mmol) and 50 mL of dry DMSO were mixed in a two-necked flask and stirred at 70 °C for 1 h. Then 4-nitrophthalonitrile (**1**, 0.30 g, 1.70 mmol) and potassium carbonate (0.18 g, 1.30 mmol) were added to the reaction mixture, after which stirring was continued for additional 7 h at a constant temperature. After a lapse of time, the reaction mixture was cooled and poured into 150 mL of a 0.1 mol/L aqueous solution of HCl and left until a precipitate formed. The precipitate was filtered off and sequentially washed with distilled water (2 × 30 mL), 0.1 mol/L HCl solution (2 × 30 mL) and distilled water (2 × 30 mL) to neutral pH, and then recrystallized from ethanol. The output was a beige fluffy powder, soluble in acetone, ethanol, chloroform, DMF. Yield: 0.26 g (82%). FT-IR: *ν*_max_, cm^−1^ 3088, 2917, 2850 (C_ar_–H); 2243 (C≡N); 1586, 1474, 1389 (C_ar_=C_ar_); 1256 (Ar–O–Ar). ^1^H-NMR (500 MHz, CDCl_3_): *δ*, ppm 7.76 (dd, 2 H, J = 8.6); 7.55 (t, 1 H, J = 8.3); 7.32 (d, 2 H, ^4^J = 2.5); 7.29 (dd, 2 H, ^4^J = 2.5, ^3^J = 8.3); 6.99 (dd, 2 H, ^3^J = 8.3, ^4^J = 2.2); 6.82 (m, 1 H, ^4^J = 2.2). ^13^C-NMR (100 MHz, acetone-d_6_): *δ*, ppm 160.66, 155.52, 135.65, 132.35, 122.03, 117.91, 117.66, 115.10, 114.74, 112.85, 109.91. MS (MALDI-TOF): *m*/*z* 362.11 [M]^+^, calcd. 363.08.

#### 3.4.2. General Route of Sandwich-Type Lanthanide Bisphthalocyaninato Synthesis

4,4’-[1,3-Phenylenebis(oxy)]diphthalonitrile (**2**, 0.10 g, 0.27 mmol) and anhydrous lanthanide acetate (0.07 mmol) were placed in a ceramic crucible and heated for about 30 min at 190 °C until the alloy was completely solidified. Rough purification was carried out by filtration on a Schott filter with chloroform to wash the alloy from impurities of the polymer composition. Next, the solvent was evaporated, and the mixture was purified by column (CHCl_3_, SiO_2_) and then gel permeation chromatography (Bio-Beads S-X1, 2.5% EtOH in CHCl_3_).

#### 3.4.3. Bistetrakis-4-[3-(3,4-Dicyanophenoxy)phenoxy]phthalocyaninato erbium (**3**)

Yield: 21%. FT-IR, *ν*_max_, cm^−1^ 3057, 2915, 2850 (C-H), 2230 (C≡N), 1593, 1481 (C=C), 1250 (Ar-O-Ar). ^1^H-NMR (500 MHz, CDCl_3_): *δ*, ppm. 7.24–6.91 (m), 6.75–6.36 (m), 3.68 (m), 2.11–1.75 (m), 1.67–1.24 (m), 0.90 (m). MS (MALDI-TOF): *m*/*z* 3065.59 [M]^+^, calcd. 3065.58. Anal. calcd for C_176_H_80_N_32_O_16_Er: C 68.95, H 2.63, N 14.62, O 8.36, Er 5.46; found: C 68.93, H 2.63, N 14.63, O 8.37, Er 5.46.

#### 3.4.4. Bistetrakis-4-[3-(3,4-dicyanophenoxy)phenoxy]phthalocyaninato ytterbium (**4**)

Yield: 20%. FT-IR, *ν*_max_, cm^−1^ 3086, 2912, 2854 (C-H), 2233 (C≡N), 1589, 1482 (C=C), 1243 (Ar-O-Ar). ^1^H-NMR (500 MHz, CDCl_3_): *δ*, ppm. 7.14–7.10 (m), 6.62–6.41 (m), 1.88 (m), 1.58–1.36 (m), 0.87 (m). MS (MALDI-TOF): *m*/*z* X [M]^+^, calcd. 3071.59. Anal. calcd for C_176_H_80_N_32_O_16_Yb: C 68.82, H 2.63, N 14.59, O 8.33, Yb 5.63; found: C 68.81, H 2.64, N 14.59, O 8.33, Yb 5.63.

#### 3.4.5. Bistetrakis-4-[3-(3,4-dicyanophenoxy)phenoxy]phthalocyaninato lutetium (**5**)

Yield: 18%. FT-IR, *ν*_max_, cm^−1^ 3092, 2915, 2856 (C-H), 2236 (C≡N), 1587, 1481 (C=C), 1239 (Ar-O-Ar). ^1^H-NMR (500 MHz, CDCl_3_): *δ*, ppm. 7.89 (s), 7.87 (s), 7.80 (s), 7.78 (s), 7.53 (t), 7.38 (dd), 7.33 (dd), 7.07 (s), 7.00 (s), 6.86 (t). MS (MALDI-TOF): *m*/*z* X [M]^+^, calcd. 3072.59. Anal. calcd for C_176_H_80_N_32_O_16_Lu: C 68.77, H 2.62, N 14.58, O 8.33, Lu 5.69; found: C 68.77, H 2.62, N 14.58, O 8.33, Lu 5.69.

## 4. Conclusions

By means of template fusion, it was possible to obtain new sandwich bisphthalocyaninates with terminal cyano- groups for a number of rare earth elements. The compounds showed outstanding spectral properties, good solubility, along with strong resistance to aggregation in chloroform and tetrahydrofuran. In acetone, atypical changes in the spectral curves were observed, apparently caused by the occurrence of equilibrium redox processes. The redox properties of the complexes were studied when neutral-radical forms of phenylhydrazine and bromine solutions were added to solutions. The rates of the processes are determined and the corresponding effective constants are calculated.

## Figures and Tables

**Table 1:** Spectroscopic characteristic of rare-earth double-decker bistetrakis-4-[3-(3,4-dicyanophenoxy)phenoxy]phthalocyanine complexes in organic solvents.

Compound	Absorption Maxima, nm (lgε)		
	Acetone	CHCl_3_	THF
bistetrakis-4-[3-(3,4-dicyanophenoxy)phenoxy]phthalocyaninato erbium (3)	670 (4.813)	673 (5.017)	670 (4.978)
		
bistetrakis-4-[3-(3,4-dicyanophenoxy)phenoxy]phthalocyaninato lutetium (4)	666 (4.806)	670 (5.053)	667 (4.908)
		
bistetrakis-4-[3-(3,4-dicyanophenoxy)phenoxy]phthalocyaninato ytterbium (5)	667 (4.714)	671 (4.947)	668 (4.802)
		

**Table 2:** Observed constants and rate constants of the redox process for solutions of macrocycles in THF in the presence of phenylhydrazine (c = 3.3 × 10^−4^ mol/L).

Macrocycle	k_obs_ × 10^3^, s^−1^
3	2.3
4	3.9
5	3.5

## Data Availability

Not applicable.

## Supplementary Materials

The following are available online, Figure S1: HR MALDI-TOF mass-spectrum of erbium (**3**) bis-tetrakis-4-[3-(3,4-dicyanophenoxy)phenoxy]phthalocyaninato (CHCA as matrix), Figure S2: HR MALDI-TOF mass-spectrum of ytterbium (**5**) bis-tetrakis-4-[3-(3,4-dicyanophenoxy)phenoxy]phthalocyaninato (CHCA as matrix). Figure S3: ^1^H NMR spectrum of lutetium (**4**) bistetrakis-4-[3-(3,4-dicyanophenoxy)phenoxy]phthalocyaninato in CDCl_3_, Figure S4: ^1^H NMR spectrum of ytterbium (**5**) bistetrakis-4-[3-(3,4-dicyanophenoxy)phenoxy]phthalocyaninato in CDCl_3_.

## Sample Availability

Samples of the compounds are not available from the authors.
