# Supplementary material for: Synthesis, Spectroscopic Properties and Redox Behavior Kinetics of Rare-Earth Bistetrakis-4-[3-(3,4-dicyanophenoxy)phenoxy]phthalocyaninato Metal Complexes with Er, Lu and Yb
Source: Molecules. 2021 Apr 10;26(8):2181. doi: 10.3390/molecules26082181 (PMC8068851; doi:10.3390/molecules26082181)
Supplement: Supplementary file 1 [file molecules-26-02181-s001.pdf]

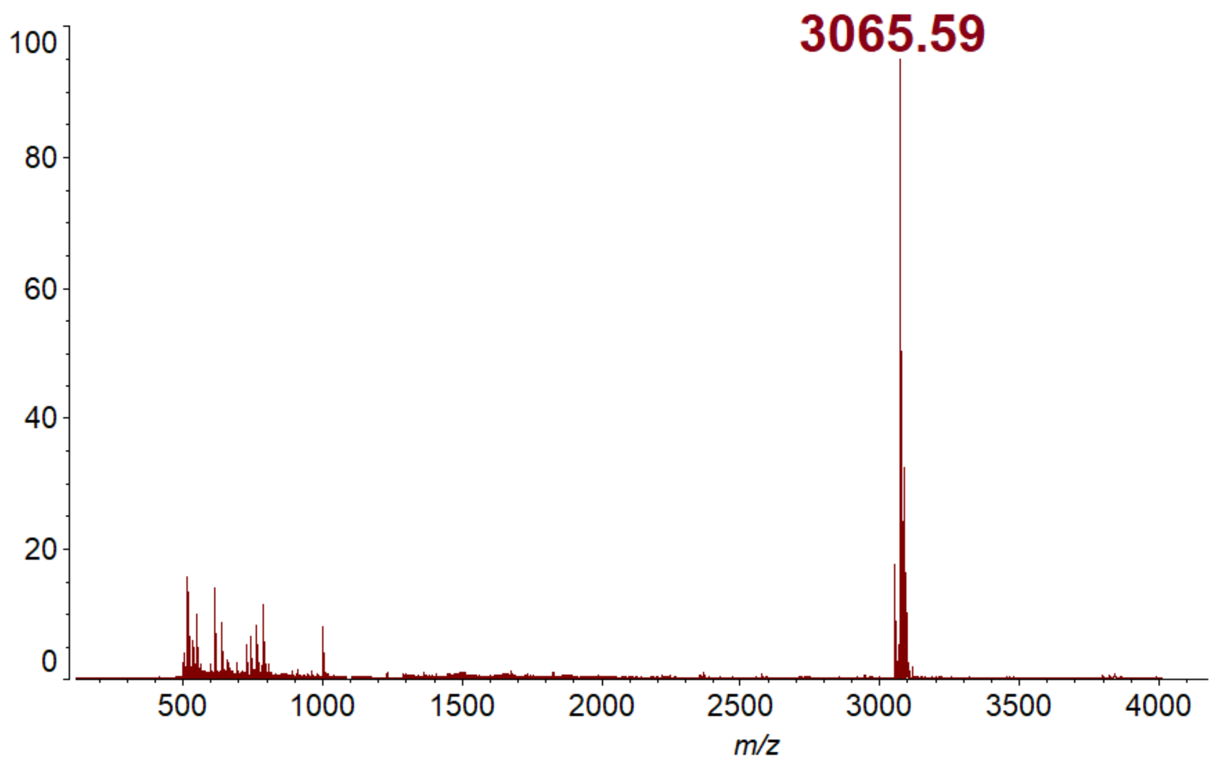

Figure S1: HR MALDI-TOF mass-spectrum of erbium (**3**) bis-tetrakis-4-[3-(3,4-dicyanophenoxy)phenoxy]phthalocyaninato (CHCA as matrix)

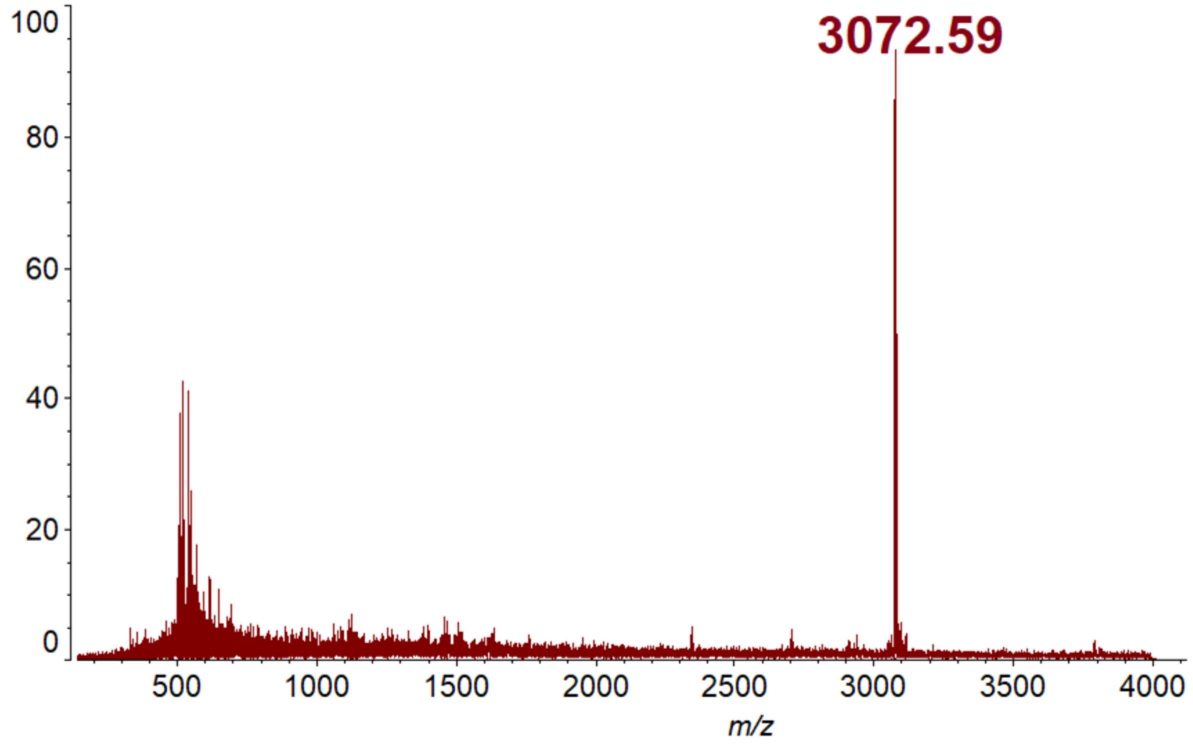

Figure S2: HR MALDI-TOF mass-spectrum of ytterbium (**5**) bis-tetrakis-4-[3-(3,4-dicyanophenoxy)phenoxy]phthalocyaninato (CHCA as matrix).

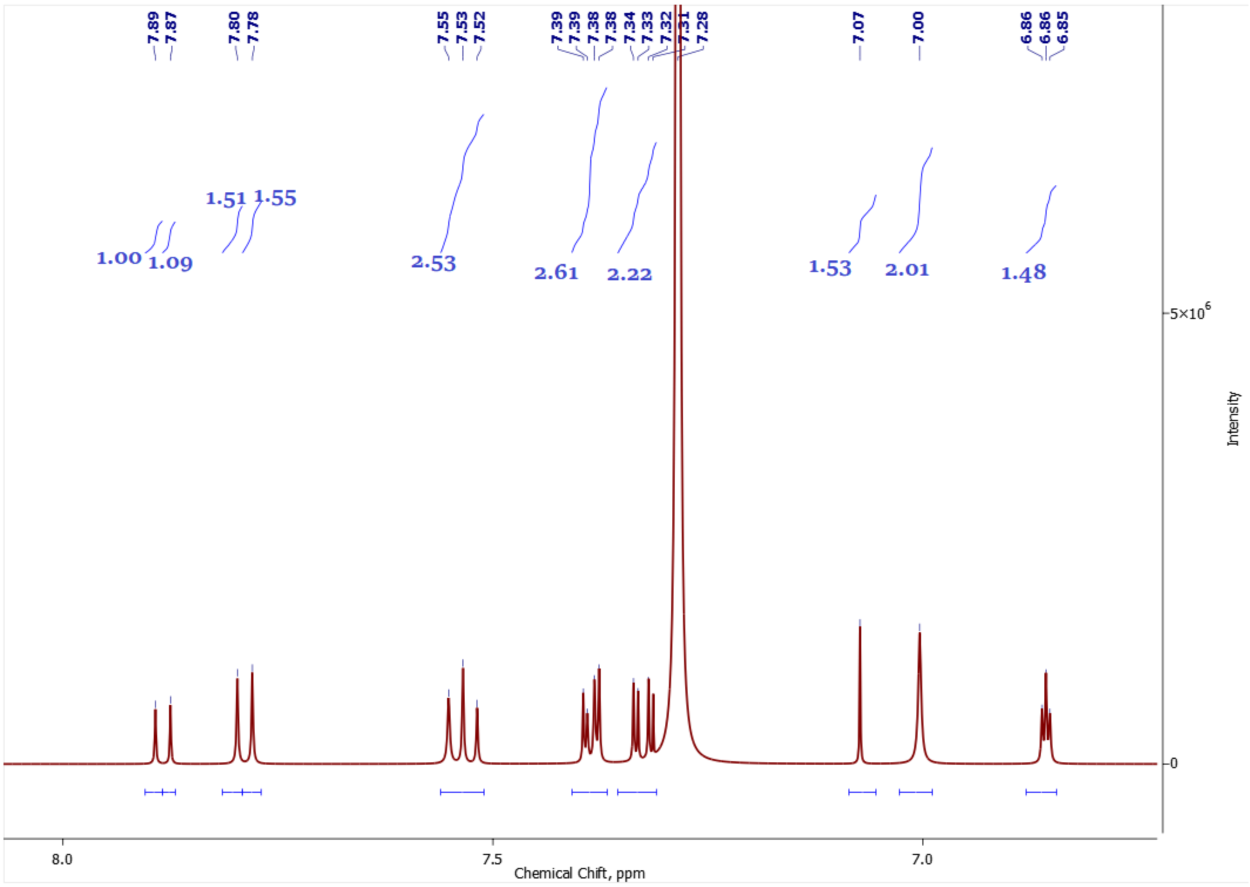

Figure S3. ^{1}H NMR spectrum of lutetium (**4**) bistetrakis-4-[3-(3,4-dicyanophenoxy)phenoxy]phthalocyaninato in $CDCl_3$,

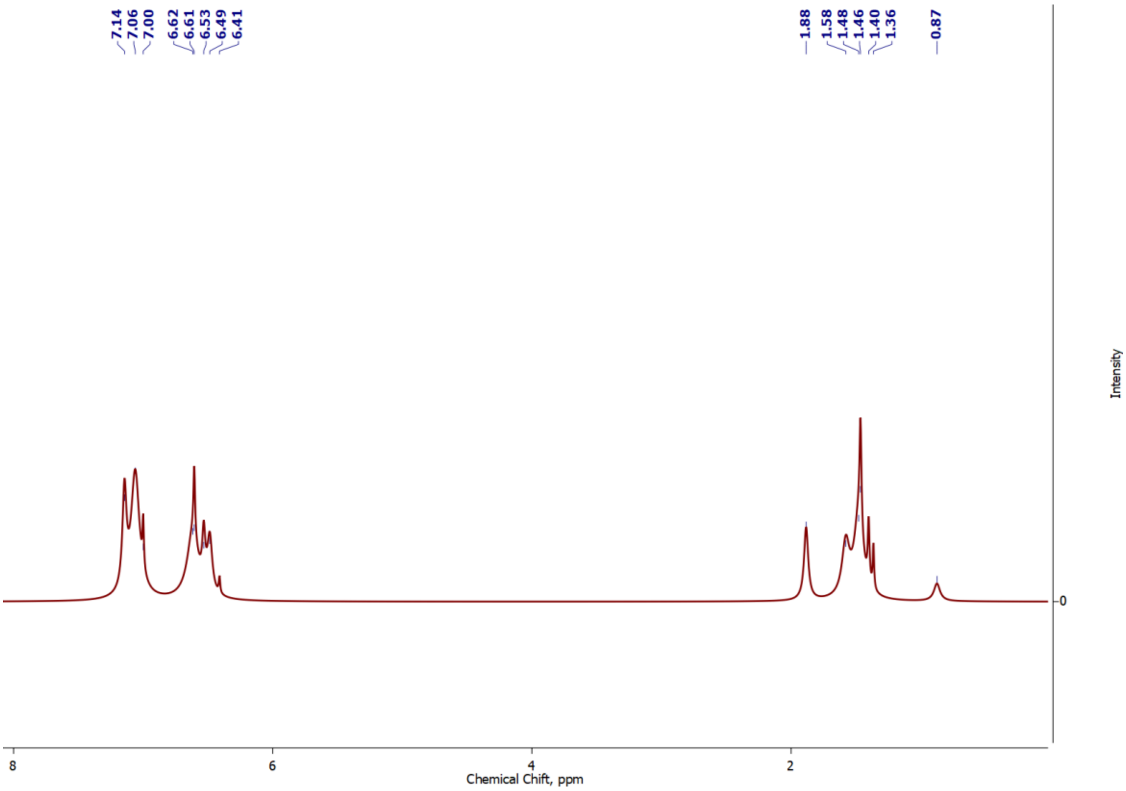

Figure S4. ^{1}H NMR spectrum of ytterbium (**5**) bistetrakis-4-[3-(3,4-dicyanophenoxy)phenoxy]phthalocyaninato in $CDCl_3$.
